# A psychophysiological investigation of laterality in human emotion elicited by pleasant and unpleasant film clips

**DOI:** 10.1186/1744-859X-9-38

**Published:** 2010-11-25

**Authors:** Hossein Kaviani, Veena Kumari, Glenn D Wilson

**Affiliations:** 1 Department of Psychology, Faculty of Health and Social Sciences, University of Bedfordshire, Luton, UK; 2 Department of Psychology, Institute of Psychiatry, King's College London, London, UK

## Abstract

**Background:**

Research on laterality in emotion suggests a dichotomy between the brain hemispheres. The present study aimed to investigate this further using a modulated startle reflex paradigm.

**Methods:**

We examined the effects of left and the right ear stimulation on the modulated startle reflex (as indexed by eyeblink magnitude, measured from the right eye) employing short (2 min) film clips to elicit emotions in 16 right-handed healthy participants. The experiment consisted of two consecutive sessions on a single occasion. The acoustic startle probes were presented monaurally to one of the ears in each session, counterbalanced across order, during the viewing of film clips.

**Results:**

The findings showed that eyeblink amplitude in relation to acoustic startle probes varied linearly, as expected, from pleasant through neutral to unpleasant film clips, but there was no interaction between monaural probe side and foreground valence.

**Conclusions:**

Our data indicate the involvement of both hemispheres when affective states, and associated startle modulations, are produced, using materials with both audio and visual properties. From a methodological viewpoint, the robustness of film clip material including audio properties might compensate for the insufficient information reaching the ipsilateral hemisphere when using static pictures. From a theoretical viewpoint, a right ear advantage for verbal processing may account for the failure to detect the expected hemispheric difference. The verbal component of the clips would have activated the left hemisphere, possibly resulting in an increased role for the left hemisphere in both positive and negative affect generation.

## Introduction

The topic of brain lateralisation, and the specialisation of the hemispheres in emotional processing and different cognitive functions involved, has been of interest to researchers in many areas and is perhaps one of the most replicated findings in the field of neuroscience [1]. Tucker and Williamson [2] concluded that the right hemisphere has a general advantage in processing emotional stimuli, whether positive or negative. However, according to some other models the right hemisphere is more involved in negative emotions [3] and the left hemisphere in positive emotions [4]. Moreover, the results of another line of research, namely the dichotic listening task, show that the right hemisphere is specialised for nonverbal tasks such as music and emotions, whereas the left hemisphere is specialised for the processing of verbal material such as words and speech [5-8].

One of the established tools for assessing emotional reactivity, which offers an interesting paradigm to probe lateralisation effects, is the modulated startle reflex [9,10]. Psychophysiological research indicates that compared to neutral conditions the startle reflex is potentiated during perception of unpleasant emotional stimuli and attenuated during perception of pleasant emotional stimuli. It has been hypothesised that the match/mismatch between the aversive properties of the startle probe and pleasant (mismatch) or unpleasant (match) nature of environmental cues (for example, images) gives rise to this linear relationship [9-12]. This effect has been observed across stimulus modalities of affective pictures (for example, [11]), sounds (for example, [12]), and odours (for example, [13,14]).

Previous studies (for example, [9,10]) investigating the impact of monaural acoustic probes administered to the left and right ears reported significant affective modulation for probes presented to the left ear, but no significant effect for probes presented to the right ear. In these studies, a set of slides with pleasant, neutral and unpleasant affective contents were used to induce different emotions. The authors speculated that the data were consistent with the notion that right hemisphere activation is dominant for affective stimuli; when startle probes were presented to the left ear (processed by right-hemisphere neural structures), larger blink amplitudes were observed in the context of foreground unpleasant stimuli in comparison with foreground pleasant stimuli.

The present study was designed to further investigate the laterality effect in human emotion by employing acoustic startle measures of emotion using an established set of affective film clips (with soundtrack) in place of slides as used by others [9-11]. Film clips produce a stronger startle modulation, indicative of a higher intensity level of emotions, than is reported generally in response to static slides [15].

## Methods

### Participants

A total of 16 right-handed volunteers (age range 18-45 years old; 8 men, mean age 29.25 years, SD = 4.41 years and 8 women, mean age 27.38 years, SD = 5.12 years) were recruited via advertisement and from an existing subject pool. They had no background of mental disorder (self-reported). Handedness was measured by actual manual performance (self-reported). The local research ethics committee approved the study procedures. All participants signed a consent form after the study procedures had been explained to them, and received £10 for their participation.

### Apparatus and materials

The film set (the same as used in the previous studies in our laboratory [15-17]) consisted of nine clips, separated by blank intervals (dark blue screen) 10-25 s long. The first three clips were used only to familiarise participants with the experimental procedure. The last six clips, used to induce emotions under experimental conditions, were presented in two blocks in the order N (neutral), P (pleasant), U (unpleasant), N, U, P. Each film clip lasted about 2 min. The set, shown using a Sharp video recorder (VC-A30HM) connected to a 20-inch Sharp colour TV monitor (DV-5101 A), was viewed from a distance of 2 m.(The supplier: Argos, London, UK).

The acoustic startle stimuli (consisting of a 50-ms presentation of a 92.5 dB (A) burst of white noise, with quasi-instantaneous rise time) were superimposed on the soundtracks (ranging from 40 to 60 dB) of the film clips, at moments of relatively low sound level, and presented monaurally via headphones (Telephonics TDH-39P) (The supplier: Argos, London, UK). During each clip, 3 startle stimuli were presented (total = 27). To increase unpredictability, they were presented with varying interstimulus intervals of 20 to 90 s after clip onset. The responses to the last 18 acoustic startle stimuli (during the last 6 clips) were included in the analyses, excluding the responses to the first 9 acoustic stimuli (during the first 3 clips, which were only for habituation).

To record electromyographic (EMG) activity of the orbicular oculi muscle, two 6 mm disc electrodes (Ag/AgCl) filled with electrolyte paste (SLE, Croydon, UK) were placed approximately 1 cm below the middle of the lower eyelid and 1 cm below the outer corner of the right eye, so that the second electrode was about 1 cm lateral and slightly higher than the first but both were parallel to the lower rim of the eyelid. An additional ground electrode was placed behind the right ear over the mastoid. Raw EMG signals were recorded, amplified, filtered, stored and analysed by a computerised startle response monitoring system (SR Instruments, San Diego, CA, USA). The analytic program treats the first 20 ms after presentation of each startle stimulus as a baseline for that trial. It then calculates latency (ms) to startle onset and peak EMG amplitude (in arbitrary analogue-to-digital units; 1 unit equals 1.2 μV, SR-Lab Program) over the 95 ms following startle onset. Trials with an unstable baseline (shift >20 units) were eliminated. Samples were taken at 1 ms (1 KHz sampling rate). The lower band pass alternative provided by the apparatus (0-500 Hz) was used throughout. The scoring criteria were identical to those used in previous studies from our laboratory [14-19]. Trials were rejected if there was evidence of excessive activity (including a premature eyeblink) during the baseline period. They were also rejected if there was no evidence of an eyeblink having been evoked by the startle probe. Altogether, 16.35% of trials were excluded on one or other of these criteria.

The affective content of each clip was rated as each clip ended (during the blank interval) on a single 11-point (-5 to +5) scale, from extremely unpleasant (for example, depressed, disgusted, angry, anxious; scored as -5), through neutral (scored as 0) to extremely pleasant (for example, happy, relaxed; scored as +5).

### Experimental design and procedure

The study consisted of two consecutive sessions, on a single occasion. The acoustic stimuli were presented monaurally to one of the ears in each session.

Participants (counterbalanced for sex) were randomly assigned in equal numbers to one of the two ear orders (left ear (session 1) - right ear (session 2); right ear (session 1) - left ear (session 2)), so that eight participants (four men and four women) received acoustic probes as well as the soundtrack of the film clips, first to the right and then to the left ear; the remaining eight participants (four men and four women) received acoustic probes first to the left and then to the right ear.

Participants were told in advance that they would be tested twice, once with left and once with right ear stimulation, while viewing a series of film clips with either pleasant, unpleasant or neutral content; that each sequence should be watched as long as it was on screen; and that throughout the experiment they would hear occasional bursts of noise through the headphones that would be neither painful nor harmful and should be ignored. The electrodes and headphones were then attached and participants were asked to keep a comfortable position in the chair while watching the video, avoiding gross body movements, and to relax, concentrate and not to attempt to control their emotions, whether positive or negative. An experimenter was present throughout the session. During each session, the affective content of each clip was rated as each clip ended (during the blank interval).

### Data reduction and analysis

The data on each of the dependent measures (affective ratings, response amplitude and latency to response onset) were separately analysed by a three-way (valence (pleasant, neutral, and unpleasant) × ear side (left and right) × ear order (left to right and right to left)) multivariate analysis of variance (MANOVA; Wilk's F), with valence and ear side as within-subjects variables and ear order as a between-subjects factor. As there were no main or interaction effects of ear order, this variable was excluded from all further analyses and the data were subjected separately to a three-way MANOVA (sex (men and women) × ear side (left and right) × valence (pleasant, neutral, and unpleasant)), with ear and valence as within-subjects variables and sex as a between-subjects factor. Since no significant main or interaction effects were found for the measures of baseline EMG and latency to response onset, only the findings on affective ratings and startle amplitude are reported here.

Although no significant interaction effect appeared in the above analysis, in order to compare the present data with that reported previously with slides ([9]; a linear trend of valence effect separately for each ear), the ear side variable was dropped from further analyses and the data for each ear separately were subjected to a two-way MANOVA (sex (men and women) × valence (pleasant, neutral, and unpleasant)), with valence as a within-subjects variable and sex as a between-subjects factor, followed by polynomial contrast tests (assessed by *t*) on valence effects.

## Results

### Affective ratings

The three-way analysis yielded no significant effects except for the main effect of valence (F(2, 13) = 70.41, *p *< 0.001).

Further analyses showed that there were significant valence effects for each ear (right ear: F(2, 13) = 59.15, *p *< 0.001; left ear: F(2, 13) = 26.54, *p *< 0.001), with highly significant linear trends (right ear: *t *= 175.787.64, *p *< 0.0001; left ear: *t *= 166.53, *p *< 0.001); a significant sex × valence effect was found for both ears: left ear, F(2, 13) = 16.50, *p *< 0.05; right ear, F(2, 13) = 24.50 *p *< 0.001. The results indicated that women found both pleasant film clips more pleasant (right ear: *t*(14) = 2.38, *p *< 0.05; left ear: *t*(14) = 2.14, *p *= 0.05) and unpleasant film clips more unpleasant than did men (right ear: *t*(14) = 2.62, *p *< 0.05; left ear: *t*(14) = 2.20, *p *< 0.05). Table 1 shows the mean affective ratings (±1 standard error of the mean) of film clips classified by ear side and sex of participants.

**Table 1:** Mean (standard error) affective ratings for men and women in right and left ear conditions.

Ear	Film clip	Men	Women
Right	Pleasant	1.31 (0.55)	2.87 (0.41)
	
	Neutral	-0.19 (0.21)	0.25 (0.21)
	
	Unpleasant	-1.63 (0.29)	-3.61 (0.41)

Left	Pleasant	1.50 (0.37)	2.88 (0.61)
	
	Neutral	0.00 (0.02)	-0.31 (0.16)
	
	Unpleasant	-1.25 (0.25)	-3.13 (0.67)

### Startle amplitude

The analyses showed significant effects for valence (F(2, 13) = 51.86, *p *< 0.001) on overall data.

Valence did not interact with sex (F(2, 13) = 0.88, *p *= 0.52) or ear side (F(2, 13) = 0.11, *p *= 0.90) There were significant valence effects for both right (F(2, 13) = 41.00, *p *< 0.001) and left (F(2, 13) = 20.21, *p *< 0.001) ears, with a significant linear effect (right ear: *t *= 33.64, *p *< 0.001; left ear: *t *= 33.64, *p *< 0.001). Table 2 presents mean startle amplitude (±1 standard error of the mean) for the two ears.

**Table 2:** Mean startle amplitude (±1 standard error of the mean) during film clips for right and left ear

Ear	Film clip	Mean (SEM)
Right	Pleasant	29.25 (4.80)
	
	Neutral	56.18 (7.64)
	
	Unpleasant	74.67 (6.08)

Left	Pleasant	24.92 (1.73)
	
	Neutral	48.84 (6.33)
	
	Unpleasant	65.20 (7.20)

## Discussion

The present study was designed to detect brain laterality effect in human emotion, using eye-blink response as a reliable component of startle reflex to a sudden loud noise, presented monaurally either to the left or right ear, modulated by the emotion-eliciting film method.

The overall data (collapsed over left and right monaural probe presentation) showed that eyeblink amplitude to acoustic startle probes varied linearly with the emotional valence of film clips. The overall affective measures over P, N, and U film clips also showed a similar linear variance. That is, P clips were rated as more positively, and U clips more negatively, relative to N clips. In addition, women rated the P conditions as more pleasant and the U conditions as more unpleasant than men. Sex has been identified as a potential factor influencing subjective ratings, that is women show more sensitivity than men to experience emotional tone in various settings, for example, during laboratory conditioning procedures [20], olfactory perception [21], and mood induction [22]. However, women might not differ from men on indices of physiological responses such as electrodermal magnitude [20].

The startle amplitude findings obtained in the present study did not show a statistically significant interaction between monaural probe side and foreground valence, suggesting no ear laterality effect in the affective modulation of the startle reflex. One reason for the observed pattern of effects may be the greater modulation of the startle responses from the film contents, which seem to be more effective in mood induction. This claim appears more logical if one takes into account some anatomical data. As pointed out by Bradley *et al*. [9], roughly two-thirds of the transmitting fibres from each ear cross the brain, and one-third proceed ipsilaterally. This means that the ipsilateral hemisphere is not completely silent while the stimulus has already activated the contralateral hemisphere. The question of 'does right ear input fail to produce the effect because the one-third of fibres directed to the ipsilateral hemisphere carry insufficient information?' was raised Bradley and colleagues. It is possible that the robustness of the film material compensates for the insufficient information. Bradley *et al*. [10], in a meta-analytic review, showed the effect sizes for the differences in reflex magnitude between pleasant and unpleasant picture categories in their previous study [9]. Overall effect sizes for the left and right ears were 0.52 and 0.17 (not significant), respectively. However, the same analysis in the present study reveals appreciably larger effect sizes for both ears (left, 0.78 and right, 0.87).

A lack of a valence × ear effect, as observed here, was also reported by Hawk and Cook [23]. In a study of the laterality of emotion, they applied tactile probes (an air puff to the side of the face) in place of acoustic probes, using the slide-viewing paradigm. Although the modulatory effect of valence was significant for tactile probes presented on the left side and not significant for probes presented to the right side, no interaction was found between valence and probe side. The latter result is in agreement with the lack of a valence × ear side effect observed in the present study.

Grillon and Davis [24] used threat of shock as an aversive context to modulate startle response. Although startle potentiation was obtained for reflexes elicited under shock threat (compared with a no-threat condition), they reported greater potentiation when startle stimuli were delivered to the right than to the left ear, implying that the left hemisphere was involved in aversive emotional processing. Similarly, in the present study, the startle amplitudes when acoustic probes were presented to the right ear in all three valences were somewhat higher than when presented to the left ear. This finding (though not significant) can be regarded as consistent with Grillon and Davis' [24] findings.

Another aspect of the present experiment that requires discussion is the possible influence of the of left hemisphere advantage for verbal material (such as words and speech) as implicated in some research findings (for example, [5,6]). Results of the dichotic task indicate the expected right ear advantage (REA) for verbal processing. We used film clips consisting of audiovisual properties that differed from the silent photographic slides used in other studies [9,10]. As a result, left-hemisphere activation would have been particularly high while participants perceived film clips as audiovisual media. In fact, the verbal component of the clips would have activated the left hemisphere, possibly giving rise to an increased role for the left hemisphere.

From a neurophysiological point of view, there are some findings that also contradict the assumption that affective modulation of startle reflex is related merely to right hemisphere processing [25]. Buchanan *et al*. [25] reported affective startle reflex modulation in a picture-viewing paradigm in control participants, but not in left or right temporal lobectomy patients. Their data as well as ours indicate the involvement of both hemispheres in affective modulation of the startle response.

Another methodological aspect of the present experiment that requires discussion is the possible influence of a central matching process in recorded eyeblink reaction. If we attribute the affect-startle effect to a central valence matching process [26], the valence match would happen within 75-ms between the probe stimulus onset and response peak. This startle timing program methodologically imposes a critical time constraint on information processing. Lang and associates [26] speculated that a startle stimulus, initiated at the right ear, fails to influence the startle modulation circuit [27] because information reaches the right hemisphere only after the motor program for the obligatory blink has been already determined. Since, in the present experiment, a 95-ms interval (during which response peak would happen) was programmed to occur after probe stimulus onset, this might allow the information to reach the right hemisphere and be processed before the activation of the obligatory blink. This feature of the experimental design may provide a neurological basis for the discrepancy between the results obtained here and those observed by others [9,10,24] in showing lateralisation of the effects of startle probes. In order to examine this possibility, in additional analyses every single startle response was manually checked and all responses measured after 75 ms (that is, when a peak occurred 75-ms after probe onset) were excluded from the data. However, we still did not find an ear × valence effect in our data. The results, moreover, showed similar patterns of response for overall valance effect and the valence effects for each ear separately.

In the present study, the EMG activity of orbicular oculi was measured only from the right eye, which precludes the possibility of investigating ipsilateral or bilateral effects of eye and ear. Thus, to fully test theories concerned with brain laterality effects on emotional responses, a further experiment is required, examining ear and eye laterality simultaneously.

To summarise, the present experiment showed the involvement of both hemispheres in affective modulation of the startle response. From methodological and theoretical viewpoints, this could be attributed to the audiovisual nature of film clips (compared to the visual nature of slides) and the right ear advantage (REA) for verbal processing.

## Competing interests

The authors declare that they have no competing interests.

## Authors' contributions

HK carried out the experiments, performed the statistical analysis and drafted the manuscript. VK and GDW participated in the design of the study, helped to interpret statistical findings and to draft the manuscript. All authors read and approved the final manuscript.
